# Mixed Management in Growing and Finishing Pigs: Impacts on Social Behavior and Judgment Bias

**DOI:** 10.3390/ani15192893

**Published:** 2025-10-03

**Authors:** Angela Cristina da Fonseca de Oliveira, Leandro Batista Costa, Saulo Henrique Weber, Antoni Dalmau

**Affiliations:** 1 Graduate Program in Animal Science, School of Medicine and Life Sciences, Pontifícia Universidade Católica do Paraná, Curitiba 80215-901, PR, Brazil; angela.fonseca@ufpr.br (A.C.d.F.d.O.); saulo.weber@pucpr.br (S.H.W.); 2 Monohub—Research Group for Monogastric Animals, Pontifícia Universidade Católica do Paraná, Curitiba 80215-901, PR, Brazil; 3 Institut de Recerca i Tecnologia Agroalimentàries—IRTA, Porcine Control and Evaluation, 17121 Monells, Spain; antoni.dalmau@irta.cat

**Keywords:** behavioral tests, cognition, emotional state, pessimistic judgment, socialization

## Abstract

Intensive pig production practices may shape cognition and behavior. We evaluated whether repeated regrouping (mixing) and gender (gilts vs. barrows) affect social interactions, fear-related responses, and affective state. A total of 96 growing pigs were separated into two treatments: control—pigs that were mixed once during the growing–finishing period; and social stress—pigs that were mixed thrice during the growing–finishing period. Social and non-social behaviors were directly noted, and four behavioral tests were conducted: open field, novel object, couples, and judgment bias tests. The statistical analysis compared gender and treatment, and *p*-values ≤ 0.05 were considered significant. Females stayed longer in the test pen entrance area during the novel object test and barrows spent more time at the feeder and defecated more during the couples’ test. With regard to the judgment bias test, females took longer to be considered trained in the discriminatory learning task and presented a “pessimistic judgment” during the ambiguous cue. Our results suggest that gender influences judgment bias in pigs and can influence social and non-social behavior, which may reflect a negative affective state with implications for their welfare and management.

## Simple Summary

Understanding how pigs respond to social and environmental challenges is essential to improve their welfare in farming systems. In this study, we investigated how mixing unfamiliar pigs and testing them in different contexts influenced their behavior and cognitive responses. Ninety-six pigs were allocated to groups, with some experiencing social stress by being mixed with unfamiliar conspecifics. We evaluated open field, food motivation, and judgment bias tasks. Gilts showed more signs of fear and caution in unfamiliar situations than barrows, taking longer to interact with food and to complete learning tasks. These results suggest that both previous social stress and sex can affect how pigs explore and make decisions. Such insights can guide improvements in welfare-oriented management on commercial farms.

## 1. Introduction

The social intelligence hypothesis posits that group living favors the development of advanced cognitive skills, because individuals must track conspecifics, identify cooperative partners, and sustain several concurrent social ties [1,2]. In such settings, animals evaluate both their own actions and those of others, as well as the outcomes of these exchanges, weighing potential costs and benefits [1]. Nevertheless, production environments in intensive systems can compromise cognition and contribute to the emergence of behavioral or health disorders [3].

Pigs are a highly social species [4,5] that use aggression to establish dominance hierarchy in the presence of unfamiliar individuals [6,7]. One particularly common concern is the frequent mixing of unfamiliar pigs on commercial farms [8]. Early weaning, which includes separation from the sow, and immature pig grouping in subsequent phases (i.e., barrows), could mean that the two most important social relationships are restricted or completely absent, making the development of stable social relationships more challenging [6]. In the field of social behavior, hormones regulate most aspects of it, from mate choices and reproduction to social cognition and aggression [9]. Social recognition is especially regulated by estrogens and evolves the ability of the animal to distinguish between conspecifics, to establish social hierarchies and social bonds. As a result, gender is presumed to be one of the most common factors that influence animals’ social structure and aggressive behavior [10].

The study of post mixing aggression is generally based on dyadic behavioral traits, which describe the direct interactions between two animals [11,12,13]. Prior research has considered the effect of mixing upon aggression, performance, and immunological traits [14,15]. However, few studies have considered gender differences during the post mixing period and its effect on other pig behavior aspects: response on fear tests (i.e., open field—OFT and novel object—NOT tests), couples, and decision-making tests (judgment bias).

Judgment bias is the propensity to judge ambiguous cues or situations optimistically [16]. It is based on the premise that subjects in a negative affective state conduct more negative judgements about ambiguous stimuli than those in a positive affective state. This type of judgment bias is generally tested in animals by training them to respond in a certain manner to a positively and a negatively associated cue (comparable behavior paradigms) [17]. Once trained, animals are exposed to intermediate, ambiguous, and novel cues [18]. Understanding how mixing management and gender can influence socialization, judgment bias, and fear discrimination in a pig can therefore be used to facilitate the improvement of housing conditions, handling routines, and farm management [19,20].

In view of the previously reported regrouping impacts [8,21], the first hypothesis of our work is that pigs who experienced social stress (mixing management) will meet training criteria slower when compared to those that were not mixed so intensively and will demonstrate a negative judgment bias against the ambiguous stimulus. Furthermore, it is expected that mixed pigs present higher agonistic interaction levels and activity during couples and fear tests (OFT and NOT), respectively. Given the role of gender in social cognition and aggression described above [9,10,11], the second hypothesis is that females will interact more positively during couples tests and will demonstrate more fear during the OFT and NOT when compared to barrows. In addition, it is expected that females will demonstrate a negative judgment bias against the neutral stimulus. Therefore, the overall objective of this study was to investigate if mixing management (non-mixed vs. mixed) and gender (gilts vs. barrows) affect socialization, fear tests, and judgment bias in pigs.

## 2. Materials and Methods

### 2.1. Ethics Declarations

All procedures complied with the national legislation and institutional standards governing animal experimentation. The experimental protocol was reviewed and authorized by the Animal Experimentation Commission of the Generalitat de Catalunya (approval no. 10329).

### 2.2. Animals and Experimental Design

A total of 96 growing pigs (48 barrows and 48 females—Duroc Commercial Line) were divided into 8 pens and separated into two treatments, with four pens per treatment. The experimental unit comprised a group of 12 animals (6 barrows and 6 females per pen), with an initial average body weight (BW) of 18.63 ± 3.05 kg and a final average BW of 129.98 ± 10.04 kg. The pigs were grouped according to body weight (penned from the lightest to the heaviest animals). The first mixing involved control and treated animals. The experiment lasted 157 days, divided into 30 days of the adaptation period and 127 days of the experimental protocol.

### 2.3. Housing Conditions

Pigs were transported from a commercial farm to the experimental facilities of IRTA, Monells, Spain (41°58′34.02″ S, 2°59′51.35″ W) and underwent a 30-day adaptation period prior to project initiation. The housing conditions and management procedures followed the EU pig standards—COUNCIL DIRECTIVE 2008/120/EC of 18 December 2008. Enrichment material (chains and wood fixed to the wall) was made available in each pen during all project phases (adaptation and growing–finishing period). The pens (5 m × 2.6 m) included a fully slatted floor, an electronic feeder system, and a nipple drinker. The floor area available to each rearing pig was > 1 m^2^. The room was climate-controlled, and the temperature was set to 19 °C ± 2 °C with a light regime set to a 12 h light–dark cycle. The pigs received feed and water ad libitum throughout the experimental period. The diet was formulated according to NRC (2012) guidelines, and all pigs received the same standardized feed throughout the study, according to the nutrient requirements for each respective period [22].

### 2.4. Treatments

The experimental treatments were as follows: T1—control group (CT): pigs that were mixed just once at the beginning of the study and not mixed again during the growing–finishing period; and T2—stress group (SS): pigs that were mixed thrice during the growing–finishing period (social stress details below).

#### Social Stress

**Figure A1:**
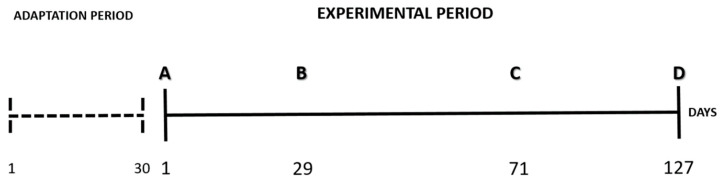
The experiment timeline. This study was conducted over 157 days, with 30 days in the adaptation period and 127 days of the experimental protocol, following the following timeline sequence: A: Initiation of the experiment and first mix conducted—distribution of control (CT) and stress (SS) groups; B: females mix—representation of the second mix conducted; C: barrows mix—representation of the third mix conducted; D: end of the experiment and pigs slaughtered.

After the initial distribution according to treatments (day 0), the SS group were mixed at two more intervals during the project. The second mix was in the fifth week of the project (day 29), and just females switched places. As all pens were half barrow and half female, during the mixing, the resident barrows remained unchanged. All females from pen number three switched places with those from pen number six. Contrarily, all females from pen number four switched places with those from pen number five. The third mix was in the eleventh week of the study (day 71), and just barrows switched places. All resident females remained unchanged. The barrows from pen number three switched places with those from pen number five. The barrows from pen number four switched places with those from pen number six. During the study, the control treatment pens remained with the animals unchanged. The study was divided into three periods for analysis of the results according to social stress as follows: period I—initiation of experimental control until the mixing of females (28 d until female mixing); period II—mixing of females until the mixing of barrows (42 d until barrow mixing); period III—mixing of barrows until the end of the experimental protocol (57 d). The experiment timeline is available in the Appendix A (Figure A1).

Pigs in the social stress treatment group were regrouped in sex batches (gilts or barrows separately). This procedure was chosen to reflect common management practices on commercial farms and to avoid potential confounds related to mixed-sex housing such as mounting behavior and sex-related growth differences. Despite retaining familiar pen-mates, regrouping still triggered agonistic interactions and hierarchy re-establishment, providing a relevant model of social stress.

### 2.5. Behavior Measures

**Figure A2:**
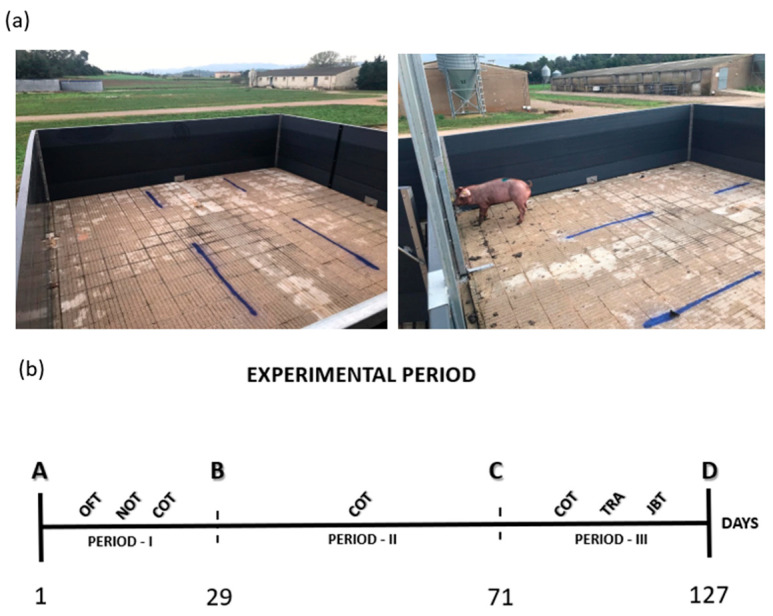
(**a**) Records of the area used to test arena and (**b**) the experiment timeline, in days, with all behavioral tests conducted by period. Symbols correspondence: OFT—open field test; NOT—novel object test; COT—couples test; TRA—training period; JBT—judgment bias test; Period I: Initiation of experimental control until the mixing of females (28 d); Period II: mixing of females until the mixing of barrows (42 d); Period III: mixing of barrows until the end of the experimental protocol (57 d).

For behavior assessments, the arena designed was located outside the shed (4.0 m × 4.0 m) in outdoor conditions without shelter, with a fully concrete floor and all sides covered with a black plastic material to block the animals’ view of the external environment. The records of the area utilized as the experimental arena, and the experiment timeline, in days, with all behavioral tests conducted, by period, are available in the Appendix A (Figure A2). The tests were conducted during the morning (0800 to 1100 h) and afternoon (1300 to 1600 h), and the animals were randomly chosen. All pigs were evaluated (the details will be described below).

The behaviors were directly noted by two observers, who were previously trained, to ensure consistency in the results. The ethograms utilized were adapted from [23,24,25] and the descriptions of each measure used are available in the Appendix A (Table A1). Another limitation of this study is that inter- and intra-observer reliability was not formally assessed during behavioral data collection. Including reliability measures would strengthen methodological rigor and should be considered in future behavioral research.

#### 2.5.1. Open Field Test (Used to Assess General Fearfulness and Exploratory Motivation) (OFT)

The OFT was conducted once during the study, in Period I. The animals were individually led to the test arena, and the measures were recorded for 2 min to assess reactivity, quadrant occupation, vocalization, and defecation behaviors. Three behavioral parameters, activity, exploration, and fear, were used to offer information about the affective states of the animals for the evaluation of reactivity.

#### 2.5.2. Novel Object Test (Used to Evaluate Neophobia and Exploratory Behavior in Response to Novelty) (NOT)

The NOT was conducted once during the study, in Period I. Previous studies have examined porcine flavor preferences [26,27] and found that sweet flavoring agents can be used for creating interest in solid food. In this test, the novel object was represented by a feeder with chopped apples. The animals were individually led to the test arena, and the following measures were recorded for 2 min: reactivity (activity, exploration, and fear), QO, vocalization and defecation behavior, and novel object manipulation. At the end of each test, the arena was briefly cleaned.

#### 2.5.3. Couples Test (Used to Assess Social Motivation and Competition over a Limited Resource) (COT)

The COT was conducted thrice during the study, in Period I, II, and III. The pigs were led to the test arena and a feeder containing chopped apples was available. The COT was conducted in pairs, and the following measures were recorded over 2 min: number of attempts to escape, defecation behavior, novel object manipulation, positive social interactions, and negative social interactions. To avoid weight and gender influence on results, the pairs had the closest weight and same gender. Once each pen had 12 growing pigs (barrows and females), six evaluations were conducted per pen, considering just 1 couple per pen.

### 2.6. Training Animals

To conduct the judgment bias test (JBT), pigs went through a week of training, from day 78 to 82 of experimentation. A barrow and a female from each pen were selected, totaling 16 animals evaluated. The barrows and females were those with the closest weight to the average weight of the pen (98.0 ± 8.0 kg). As the training period lasted one week, the authors always changed the group of animals trained by period (morning and afternoon), preventing the same group from being trained only during the morning or only during the afternoon.

**Figure 1:**
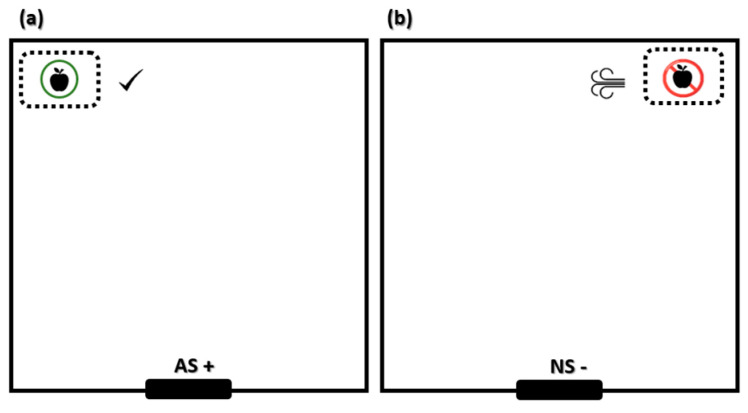
Scheme with the distribution of feeders for animal training. (**a**) Allowed scenario (AS+). The pig finds a feeder positioned on the right corner of the test arena containing chopped apples and is allowed to pick up the apples. (**b**) Non-allowed scenario (NS−). The pig finds a feeder positioned on the left corner of the arena (opposite side of AS+), also containing chopped apples. When the pig approaches the feeder on the left limb, it receives a punishment represented by a blast of air followed by a loud noise (approximately 90 Db for 5 s).

The training comprised pigs being able to distinguish, depending on feeder position, when they were allowed to eat the apples and when they were not allowed (Figure 1). In the “allowed scenario” (AS+), the pigs found a feeder positioned on one corner of the arena containing chopped apples. As the pig approached the feeder it was allowed to eat the apples. Each correct ‘go’ response to the AS+ was rewarded by allowing the pig to eat the apples for 1 min, whilst a ‘no-go’ response to the AS+ was not rewarded. The pig was simply removed from the arena after 1 min and returned to the pen. In the “non-allowed scenario” (NS−), pigs found a feeder positioned on the other corner of the arena (opposite side of AS+), also containing chopped apples. When pigs approached the feeder they received a punishment and were not allowed to eat the apples. The punishment was a blast of air at high pressure, close to the snout, immediately followed by a loud noise. The blast of air was applied with an air compressor (50 L, 2 Hp, Cosmos Industrial, Barcelona, Spain) and the noise (approximately 90 Db during 5 s) was a result of the compressor engine. Pigs’ hearing range is similar to that of humans, but with a shift towards ultrasound. The frequency range for reasonable detection varies between 42 Hz and 40.5 kHz, with a region of best sensitivity from 250 Hz to 16 kHz [28] and a demonstrated aversion to sudden loud noise with an intensity of 85–97 dB [29]. After the punishment, one of the observers entered the arena and gently led the animal to the pen. Each correct ‘no-go’ response to the NS− was not rewarded or punished. If the pig did not try to approach the feeder for 1 min, it was removed from the arena. For both positive and negative scenarios, the training session lasted for 1 min.

Half of the animals (*n* = 8) started training for AS+, and the other half started training with NS−. All animals selected were trained in both scenarios. To avoid a “side effect”, that is, to ensure that the behaviors observed are not only due to the feeder position, but due to the learning of the animals, half of the animals (4 barrows and 4 females) were trained with AS+ (allowed to eat apples) on the right side of the test arena and NS− (punishment) on the left side. The other half of the animals were trained with AS+ on the left side and the NS− on the right side. The total number of training sessions required for each animal and the time spent were recorded. The pig was considered trained when, during three consecutive tests, it presented the expected behavior according to the feeder position. AS+: Ate the apples when allowed, or NS−: during the training session (1 min), did not approach the feeder when the position indicated a punishment. Two females (one from the CT group and one from the SS group) were removed from the study because they did not learn the task. After the first training followed by punishment, these females no longer entered the testing arena.

#### Reminder Sessions and Judgment Bias Test

**Figure 2:**
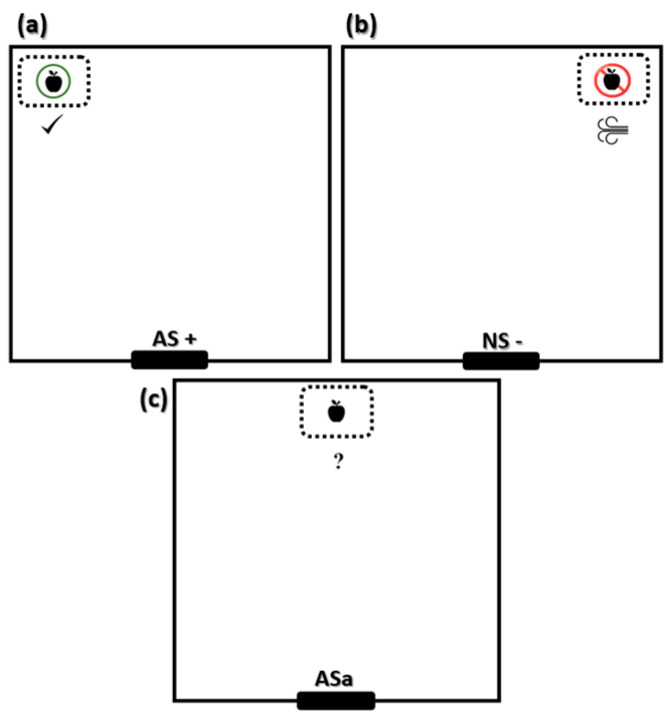
Scheme with feeder distribution for the reminder sessions and the judgment bias test (**a**) allowed scenario (AS+); (**b**) non-allowed scenario (NS−); (**c**) ambiguous stimulus (ASa).

After a week of training, pigs underwent a reminder session (day 85). One additional AS+ and NS− reminder session was conducted before the judgment bias session. The latency to contact the feeder, defined as the time pigs took from entering the test arena to the contact with the chopped apples, was recorded in all sessions. After the end of the reminder session (day 85), the judgment bias test was applied 24 h later (day 86). The trials ran from 0800 until 1130 h in the morning and from 1300 until 1600 h in the afternoon. We tested all 16 pigs on the same day, 8 in the morning and 8 in the afternoon. In the judgment bias tests (JBT), an ‘ambiguous’ (ASa) scenario was presented to investigate whether animals responded to this cue as predicting the reward (‘optimistic’ response) or punishment (‘pessimistic’ response) [30]. Since ambiguity for pigs is short-lived [31], each pig was evaluated only once for the ambiguous stimulus (ASa). A feeder containing chopped apples was positioned in the center of the test arena, in an ambiguous position (Figure 2). The JBT finished when the pig ate the chopped apples or 60 s after entering the test pen. At the end of the JBT, the test pen door was opened, and the pig returned to the housing pen. The approach behavior of the pig (latency to touch the object) was recorded. To assess fear, other measures were also recorded during JBT, such as escape attempts, QO, vocalization and defecation behavior, and feeder interaction (time spent touching the new object).

### 2.7. Statistical Analysis

All analyses were performed in STATGRAPHICS Centurion XVI (v. 16.11). Treatment (CT vs. SS) and gender (barrow vs. gilt) were used as fixed factors. Data from parametric variables were analyzed by GLM and compared by analysis of variance (ANOVA-Type III) followed by Tukey’s test when homogeneity of variance was observed (Levene’s test), otherwise the Bonferroni test was applied. When the data did not demonstrate normality (Shapiro–Wilk) or homogeneity of variance (Levene), a Wilcoxon test was conducted. The results are presented as the mean and standard error. Data from the reminder sessions and judgment bias test were analyzed using a GLM with repeated measures followed by a post hoc Tukey’s test. We compared the latency for each animal reaching the food bowl within 60 s (‘go’ response) for each cue type (AS+, NS−, ASa). Two statistical analyses were utilized for non-parametric data, depending on the type and variation in the data obtained. The results of the frequency analysis, with a vast variation range, included the following: QO, number of vocalizations, number of training sessions for negative stimulus, and number of training sessions for positive stimulus. These were compared by the Mann–Whitney (Wilcoxon) W-test. Nonparametric data from binary analyses (yes/no) were compared by Kendall’s Tau-b (τb) correlation coefficient. All the non-parametric data are presented as the medians and minimum–maximum values, and the description of each test used is indicated in the tables. *p*-values ≤ 0.05 were considered significant.

Given the sample size and distributional properties of several variables, we used a combination of GLM/ANOVA and non-parametric tests as appropriate. We acknowledge that mixed-effects or Bayesian models could capture additional variance components and complex factor structures; adopting such approaches is a valuable avenue for future studies with larger samples and repeated measures.

## 3. Results

### 3.1. Open Field (OFT) and Novel Object Test (NOT)

Data from the OFT and NOT are represented in Table 1 and Table 2, respectively. In the OFT, no gender-related differences were observed (Table 1). In the NOT, however, females spent more time in the entry and central quadrants (QO1 and QO2) compared with barrows (*p* ≤ 0.05; Table 2).

### 3.2. Couples Test

Data from the couples test according to treatment and gender are represented in Table 3. During Period I, no treatment effects were detected. In Periods II and III, pigs in the SS group spent more time at the feeder, and in Period II they also defecated more frequently (*p* ≤ 0.05). For the differences between gender, barrows spent more time at the feeder during Period III and defecated more during Period I when compared to females (*p* ≤ 0.05).

### 3.3. Animal Training

Data from the training sessions are represented in Table 4. There was no statistical difference between treatment (CT vs. SS) for the variables of global training time for non-allowed scenario (TNS−), global training time for allowed scenario (TAS+), number of training sessions for non-allowed scenario (NNS−), and number of training sessions for allowed scenario (NAS+). However, females required significantly longer to achieve the TAS+ criterion (1295.13 s) compared with barrows (343.50 s; *p* ≤ 0.05). There was no statistical difference for the variables TNS−, NNS−, and NAS+.

### 3.4. Judgment Bias Test

**Figure 3:**
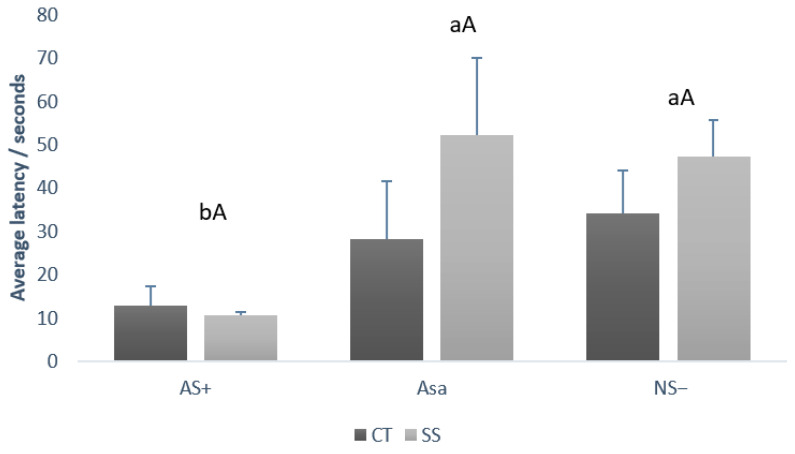
Comparison between the average latency to touch the feeder (+SE), of CT and SS growing–finishing pigs during judgment bias test, by each cue type (AS+, NS−, and ASa). Lowercase letters (ab) compare latency between cue type (AS+ vs. NS− vs. ASa) regardless of treatment. Capital letters (A) compare latency between treatments (CT vs. SS) within each cue type. CT: Pigs that were mixed once during the growing–finishing period; SS: pigs that were mixed three times during the growing–finishing period.

**Figure 4:**
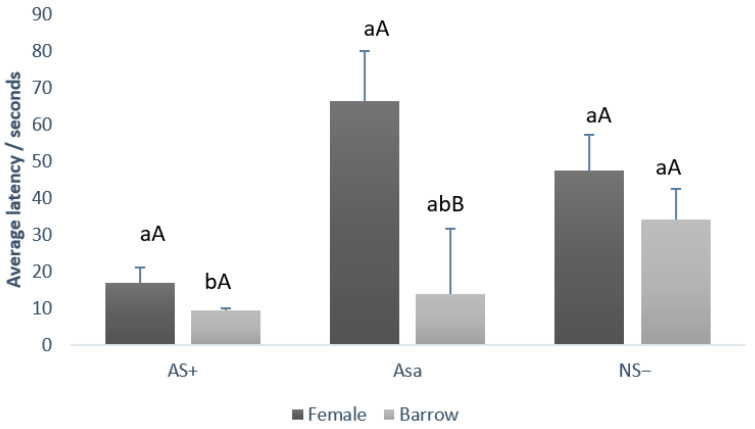
Comparison between the average latency to touch the feeder (+SE), of barrows and female growing–finishing pigs during judgment bias test by each cue type (AS+, NS−, and ASa). Lowercase letters (ab) compare latency between cue type (AS+ vs. NS− vs. ASa) in each gender. Capital letters (AB) compare latency between gender (barrow vs. female) within each cue type.

Data from the judgment bias test according to treatment and gender are depicted in Figure 3 and Figure 4, respectively. There was no statistical difference between treatments (CT vs. SS) by cue type (capital letters, *p* = 0.178). Comparing the average latency independent of the treatment, it was possible to observe that the pigs presented a lower latency (lowercase letters, *p* = 0.034) for the AS+ (11.75 ± 2.56 s) cue type when compared to NS− (40.75 ± 9.07 s) and ASa (40.12 ± 15.65 s).

Regarding the comparison between gender, barrows and females demonstrated the same latency (*p* > 0.05) to touch the feeder during AS+ (barrow 9.25 ± 0.72 s and female 16.75 ± 5.11 s) and during the NS− (barrow 34.00 ± 9.87 s and female 47.5 ± 8.23 s). However, for the ambiguous cue (ASa), females required significantly longer to approach the feeder (66.5 ± 18.09 s) compared with barrows (13.75 ± 3.75 s; *p* = 0.001). Furthermore, comparing latency between cue type (AS+ vs. NS− vs. ASa) in each gender, it was observed that females presented the same latency (*p* > 0.05) between the three cue types whereas barrows demonstrate some differences (lowercase letters, *p* = 0.001). Barrows demonstrate lower latency values during AS+ when compared to NS−, and intermediary values during ASa.

## 4. Discussion

No differences were found between gender in the OFT. Females remained longer in the pen entrance quadrants (higher QO1 and QO2 score) when compared to barrows in the NOT. A possible explanation for these results is fear, or more careful behavior, from females in the face of unfamiliar situations when compared to barrows. Some authors investigated possible differences between genders in social behavior and cognition regulation [9,32,33]. The typical and expected porcine response to a new environment with the offer of a different food that arouses their interest is to spend more time manipulating the food, but it is not certain whether this behavior reflects feeding or exploratory motivation or a combination of both [34]. From an evolutionary perspective, it has been speculated that the male superiority in spatial ability would be more prominent when there was pressure for males to traverse larger areas to gain access to mates [35]. Furthermore, some studies describe the difference between the genders regarding group formation and gregarious behavior in pigs, which can interfere in the responses of animals when exposed to new situations individually, away from the group [36,37]. According to [38], the gregarious structure is more prevalent among females while solitary behavior is more prevalent among males. This fact could elucidate the QO differences during NOT and the apparent retraction of females moving within the arena when separated from the group. Some findings also suggest that other underlying neurochemical mechanisms to support social learning may differ between the genders [39,40]. Some authors found that males castrated neonatally are generally more active than entire males or males castrated at 30 days of age [41,42]. Whether androgens or estrogens similarly mediate social learning in barrows is still obscure [9,34]. The same pattern of female behavior in the face of novel situations was observed during the discriminatory learning task and judgment bias test, which will be discussed in the subsequent paragraphs. More studies investigating the differences between gender (male vs. female) and between intact males and castrated males (barrows) should be conducted. Understanding the nuances between genders can be a tool for producers facing intensive swine production management.

According to the couples test, no differences were found between the variables in Period I during the analysis by treatment, which was expected since all animals underwent the same relocation condition. The premise of “resource holding potential” (RHP) refers to the ability of an individual to compete for and retain the resources required for survival and reproduction [43], represented by their body size or weight [44,45]. Considering RHP, all pairs formed for the couples test considered the sex (individuals of the same sex) and weight (inside the pen, individuals with similar weights) of the animal. The objective of this methodology was to avoid the effect of the above RHP test results, keeping only the effect of the mix.

The SS group couples spent more time at the feeder during Period II and III and defecated more during Period II. Considering the disputes over hierarchy, and also the places with the highest number of agonistic interactions within the pen, it can be considered that mixing keeps the animals more avid for the limited resource, which may elucidate the fact that the SS group animals remained longer at the feeder when compared to the CT group. For the differences between gender, barrows spent more time at the feeder during Period III when compared to females. For these results, the same hypothesis regarding limited resource disputes can be raised, considering that the pairs were formed based on sex and that, in period III, the mix was conducted with barrows, the gender that remained the longest at the feeder.

Considering previous work on resource competition, some of the important environmental factors influencing defensibility are the crowd or agglomeration of resources in space and time, the predictability of their arrival in space and time, and the number of potential competitors present [46]. Despite being an item of high interest to the animals and offered in a limited manner, the couples test was not the first test to be conducted with these animals using apples, which could have reduced their perception of them as a limited resource. Also, the number of competitors (pair evaluation) may have influenced the absence of agonistic interactions, considering that only two animals may not represent a great threat to the resource in question; and since the mixings were made by sex, the pairs formed contained animals who had been pen mates since the study initiation. Although the control and the treated groups demonstrated very similar results, more studies to elucidate the effects of social stress on division and competition for resources need to be conducted. Future studies should examine whether mixing strategies act as social support during relocation or merely intensify hierarchical disputes.

Contradicting the initial hypothesis, it was not possible to detect statistical differences between treatments (CT vs. SS) during the animal training sessions (TNS−, TAS+, NNS−, and NAS+ variables). However, evaluating the isolated effect of gender, it was observed that females took longer to be considered trained in the discriminatory learning task AS+ (1295.13 s) when compared to barrows (343.50 s). After going through the negative cue (NS−), females reduced their intensity of access or approaching the feeder with the reward (AS+), taking a long time to reach the necessary criteria. These results verify previous findings indicating gender differences in the inhibition of “learned fear”. More recent animal studies have begun to redress this imbalance by examining sex differences in the regulation of learned fear using translationally relevant behavioral paradigms [33]. Animal research has also demonstrated impaired fear extinction processing in females and these studies are beginning to elucidate the underpinning neural circuit, neurochemical, and endocrine mechanisms [47,48,49]. In a study conducted by [33], males demonstrated marginal fear discrimination after limited training and successful discrimination with extended training. Conversely, females displayed fear discrimination with limited training and generalization post-extended training. One possibility is that the stressful experience of the first day of training affected subsequent discrimination learning differently in barrows and females over the following training day(s). However, sex differences in fear discrimination involving discrete cues and the role of altered safety signaling in mediating these differences are obscure.

The same fearful behavior was observed in the judgment bias test. The initial hypothesis of our study was that animals that underwent stress would present a negative judgment bias against the neutral stimulus, represented by the feeder in the center. According to the results, it was possible to observe that all animals (CT and SS group) presented the same judgment about the ambiguous cue Asa as they did with NS−, demonstrating similar latency to touch the feeder. Females presented a “pessimistic judgment” during the ambiguous cue when compared to barrows, as they took longer to approach and touch the feeder as compared to AS+. Fear discrimination and safety signaling both depend on estrogen receptor signaling in females, and evidence indicates that sex differences in contextual fear discrimination also depend on estrogen [50,51]. From an adaptive perspective, there might be different circumstances which favor discrimination or generalization in relation to salient stimuli. Rapid discrimination between threat-related and harmless stimuli may conserve resources by restricting appropriate behavioral responses to a limited number of cues. Conversely, generalizing across cues may enhance survival by promoting defensive responses to a wider range of stimuli that potentially predict threat, perhaps under more uncertain or stressful environmental conditions [33].

Considering the time spent in training sessions, and the overall project duration, it was not possible to increase the N for the number of animals evaluated in the judgment bias test, which may have contributed to the absence of statistical differences observed in some results. For future research, it would be interesting to repeat the tests conducted investigating the differences between treatment and gender for a larger number of animals.

## 5. Conclusions

Repeated mixing influenced pigs’ social behavior during the couples test, with mixed animals spending longer at the feeder than controls. Regarding sex, gilts remained longer near the entrance during the novel object test, while barrows spent more time at the feeder and defecated more in the couples test. In the judgment bias assessment, gilts took longer to meet the discrimination criterion and showed a more pessimistic response to the ambiguous cue than barrows. These findings indicate that management related to regrouping and biological sex shape social and non-social behaviors and cognitive judgments in growing–finishing pigs, with direct implications for housing and handling practices.

## Figures and Tables

**Table 1:** Data from the open field test according to the growing–finishing pigs gender.

Item	Gender		*p*-Value
	Female	Barrow	
^1^ One-Way ANOVA			
Activity (s)	88.06 ± 9.72	70.59 ± 9.93	0.212
^2^ Mann–Whitney: W-Test			
Reactivity (n)	0 (0–1)	0 (0–2)	0.712
QO—1 (n)	3 (1–7)	3 (1–8)	0.391
QO—2 (n)	5 (2–14)	4.5 (1–13)	0.432
QO—3 (n)	3 (0–7)	2 (0–6)	0.883
Vocalization (n)	15.5 (0–79)	15.5 (0–61)	0.997
^3^ Kendall’s Tau b			
Defecation (%)	34.04	37.23	0.316

QO—1: quadrant for animals entering the arena; QO—2: central quadrant, where the feeders were positioned; QO—3: quadrant located laterally to the corral and opposite the entrance to the arena. ^1^ Parametric data presented as mean ± standard error and compared by ANOVA-Type III followed by Tukey’s test when homogeneity of variance (Levene’s test) was observed. ^2^ Nonparametric data resulting from scores presented as median (minimum–maximum) and compared by Mann–Whitney (Wilcoxon) W-test to compare the medians of the two samples. ^3^ Nonparametric data resulting from binary data (presence/absence) are presented at percentage and compared by Kendall’s Tau b-test to compare count and frequency.

**Table 2:** Data from novel object test according to growing–finishing pigs gender.

Item	Gender		*p*-Value
	Female	Barrow	
^1^ One-Way ANOVA			
Latency (s)	53.51 ± 6.34	63.60 ± 6.62	0.274
Duration (s)	30.10 ± 4.26	23.91 ± 4.45	0.317
^2^ Mann–Whitney: W-Test			
Reactivity (n)	0 (0–2)	0 (0–1)	0.501
QO—1 (n)	3 (1–10) a	2 (1–6) b	0.003
QO—2 (n)	5 (1–14) a	3 (1–12) b	0.050
QO—3 (n)	2 (0–6)	2 (0–6)	0.330
Vocalization (n)	15 (0–74)	14 (0–47)	0.609
^3^ Kendall’s Tau b			
Defecate (%)	17.02	20.21	0.340

QO—1: quadrant for animals entering the arena; QO—2: central quadrant, where the feeders were positioned; QO—3: quadrant located laterally to the corral and opposite the entrance to the arena. ^1^ Parametric data presented as mean ± standard error and compared by ANOVA-Type III followed by Tukey’s test when homogeneity of variance (Levene’s test) was observed. ^2^ Nonparametric data resulting from scores presented as median (minimum–maximum) and compared by Mann–Whitney (Wilcoxon) W-test to compare the medians of the two samples. *p* ≤ 0.05 at 95.0% confidence level. ^3^ Nonparametric data resulting from binary data (presence/absence) are presented as percentage and compared by Kendall’s Tau b—test to compare count and frequency; a,b Different letters on the row compare gender by application site and represent differences between medians by Mann–Whitney (Wilcoxon) W-test (*p* ≤ 0.05).

**Table 3:** Average data of couples test according to growing and finishing pigs’ treatment and gender.

Item	Period	Treatment			Gender		
		CT	SS	*p*-Value	Female	Barrow	*p*-Value
^1^ One-Way ANOVA							
Latency (s)							
	I	18.08 ± 4.74	26.15 ± 4.74	0.232	17.46 ± 4.73	26.77 ± 4.73	0.167
	II	28.61 ± 4.61	24.42 ± 4.51	0.517	21.22 ± 4.55	31.50 ± 4.46	0.110
	III	35.38 ± 5.45	29.48 ± 5.45	0.447	37.13 ± 5.43	27.73 ± 5.43	0.224
Duration (s)							
	I	39.13 ± 4.14	42.38 ± 4.14	0.580	43.98 ± 4.12	37.52 ± 4.12	0.271
	II	24.11 ± 3.26 b	36.00 ± 3.19 a	0.011	31.85 ± 3.37	28.58 ± 3.30	0.491
	III	20.79 ± 3.23 b	33.00 ± 3.23 a	0.009	20.50 ± 3.22 B	33.29 ± 3.22 A	0.006
^2^ Mann–Whitney: W-Test							
Reactivity (n)							
	I	0 (0–1)	0 (0–0)	0.327	0 (0–0)	0 (0–1)	0.327
	II	0 (0–3)	0 (0–1)	0.976	0 (0–3)	0 (0–0)	0.151
	III	0 (0–0)	0 (0–1)	0.327	0 (0–1)	0 (0–0)	0.327
^3^ Kendall’s Tau b							
Defecate (%)							
	I	7.29	14.58	0.086	6.25 Y	15.36 X	0.027
	II	2.13 y	11.7 x	0.010	4.26	9.57	0.160
	III	1.04	1.04	1.000	2.08	0.00	0.155

CT: Pigs that were mixed once during the growing–finishing period; SS: pigs that were mixed three times during the growing–finishing period. Period: I—Start of experimental control until the mixing of females (28 d); II—mixing of females until the mixing of barrows (42 d); III—mixing of barrows until the end of the experimental protocol (57 d). ^1^ Parametric data presented as mean ± standard error and compared by ANOVA-Type III followed by Tukey’s test when homogeneity of variance (Levene’s test) was observed. ^2^ Nonparametric data resulting from scores presented as median (minimum–maximum) and compared by the Mann–Whitney (Wilcoxon) W-test to compare the medians of the two samples. *p* ≤ 0.05 at 95.0% confidence level. ^3^ Nonparametric data resulting from binary data (presence/absence) are presented as percentage and compared by Kendall’s Tau b-test to compare count and frequency. *p* ≤ 0.05 at 95.0% confidence level. a,b Different letters on the row compare treatment and represent differences between means by ANOVA followed by Tukey’s test (*p* ≤ 0.05). A,B Different letters on the row compare gender and represent differences between means by ANOVA followed by Tukey’s test (*p* ≤ 0.05). x,y Different letters on the row compare treatment and represent differences between frequency by Kendall’s Tau b (*p* ≤ 0.05). X,Y Different letters on the row compare gender and represent differences between frequency by Kendall’s Tau b (*p* ≤ 0.05).

**Table 4:** Total training time (mean ± SE) and training session number (median/min–max) for discriminatory learning task, with positive and negative stimulus, in finishing pigs.

Item	-	Treatment			Gender		
		CT	SS	*p*-Value	Female	Barrow	*p*-Value
^1^ One-Way ANOVA							
TNS−	s	497.50 ± 79.13	624.00 ± 79.13	0.280	582.13 ± 79.13	539.38 ± 79.13	0.709
TAS+	s	775.00 ± 227.65	863.625 ± 227.65	0.788	1295.13 ± 227.65 a	343.50 ± 227.65 b	0.012
^2^ Mann–Whitney: W-Test							
NNS−	n	13 (9–34)	13 (9–26)	0.705	13 (9–34)	13.5 (9–26)	1.000
NAS+	n	19 (9–23)	15.50 (9–26)	1.000	21 (9–26)	13.50 (9–22)	0.126

CT: Pigs that were mixed once during the growing–finishing period; SS: pigs that were mixed three times during the growing–finishing period. TNS−: Global training time for non-allowed scenario; TAS+: global training time for allowed scenario; NNS−: number of training sessions for non-allowed scenario; NAS+: number of training sessions for allowed scenario. ^1^ Parametric data presented as mean ± standard error and compared by ANOVA-Type III followed by Tukey’s test when homogeneity of variance (Levene’s test) was observed. *p* ≤ 0.05 at 95.0% confidence level. ^2^ Nonparametric data resulting from scores presented as median (minimum–maximum) and compared by Mann–Whitney (Wilcoxon) W-test to compare the medians of the two samples. *p* ≤ 0.05 at 95.0% confidence level. a,b Different letters on the row compare gender and represent differences between means by ANOVA followed by Tukey’s test (*p* ≤ 0.05).

## Data Availability

The datasets generated and/or analyzed during our study are available from the corresponding author upon reasonable request.

## Abbreviations

The following abbreviations are used in this manuscript:

PUCPR	Pontifícia Universidade Católica do Paraná
IRTA	Institut de Recerca i Tecnologia Agroalimentàries
EU	European Union
OFT	Open Field Test
NOT	Novel Object Test
COT	Couples Test
JBT	Judgment Bias Test
AS+	Allowed Scenario (Positive)
NS−	Non-Allowed Scenario (Negative)
ASa	Ambiguous Stimulus (Ambiguous Scenario)
QO	Quadrant Occupation
CT	Control Treatment
SS	Social Stress
BW	Body Weight
GLM	General Linear Model
ANOVA	Analysis of Variance
TNS−	Training Time for Non-Allowed Scenario
TAS+	Training Time for Allowed Scenario
NNS−	Number of Training sessions for Non-Allowed Scenario
NAS+	Number of Training sessions for Allowed Scenario

## Appendix A

**Table A1:** Ethogram of measures for tests conducted during the experimental period.

Measure		Description	Behavioral Tests ^b^
Reactivity	Activity	- No movement of any portion of the pig body was visible for a period > 2 s. The duration includes from the beginning of the freezing/immobility until any body movement.	OFT, NOT, JBT.
		- No movement of any portion of the pig body was visible during the entire testing period (freezing).	
	Exploration	- Time moving at least three feet (seconds/minute).	OFT, NOT, JBT.
		- Time spent manipulating/exploring the floor or walls with the nose (seconds).	
	Fear	- Number of attempts to escape/raising front legs against wall (seconds).	OFT, NOT, COT, JBT.
Quadrant occupation		- Number of times that animals crossed a quadrant line with both front legs.	OFT, NOT, JBT.
Vocalization		- Number of vocalizations.	OFT, NOT, JBT.
Eliminatory Conducts		- Defecation (yes/no).	OFT, NOT, COT, JBT.
Novel object manipulation		- Latency to touch the feeder with apples (seconds).	NOT, COT, JBT.
		- Time spent manipulating/touching the apples (seconds).	
Social interactions		- Number of positive interactions: playing, nosing, non-agonistic physical contact.	COT.
		- Number of negative interactions: reciprocal fighting, attacking, biting, head knocking, inverse pressing, parallel pressing.	

^b^ OFT: open field test; NOT: novel object test; COT: couples test; JBT: judgment bias test.
